# Supplementary material for: Dolphin Pituitary Gland: Immunohistochemistry and Ultrastructural Cell Characterization Following a Novel Anatomical Dissection Protocol and Non-Invasive Imaging (MRI)
Source: Animals (Basel). 2025 Mar 4;15(5):735. doi: 10.3390/ani15050735 (PMC11898967; doi:10.3390/ani15050735)
Supplement: Supplementary file 1 [file animals-15-00735-s001.zip › animals-3481132-supplementary.pdf]

Supplementary information for:

Dolphin Pituitary Gland: Immunohistochemistry and Ultrastructural cell characterization following a novel anatomical dissection protocol and non-invasive imaging (MRI).

Paula Alonso-Almorox [1,2], Alfonso Blanco [2] Carla Fiorito [3], Eva Sierra [1], Cristian Suárez-Santana [1], Francesco Consolli [1], Manuel Arbelo [1], Raiden Grandía Guzmán [1], Ignacio Molpeceres-Diego [1], Antonio Fernández Gómez[5], Javier Almunia [4], Ayoze Castro [1] and Antonio Fernández [1*]

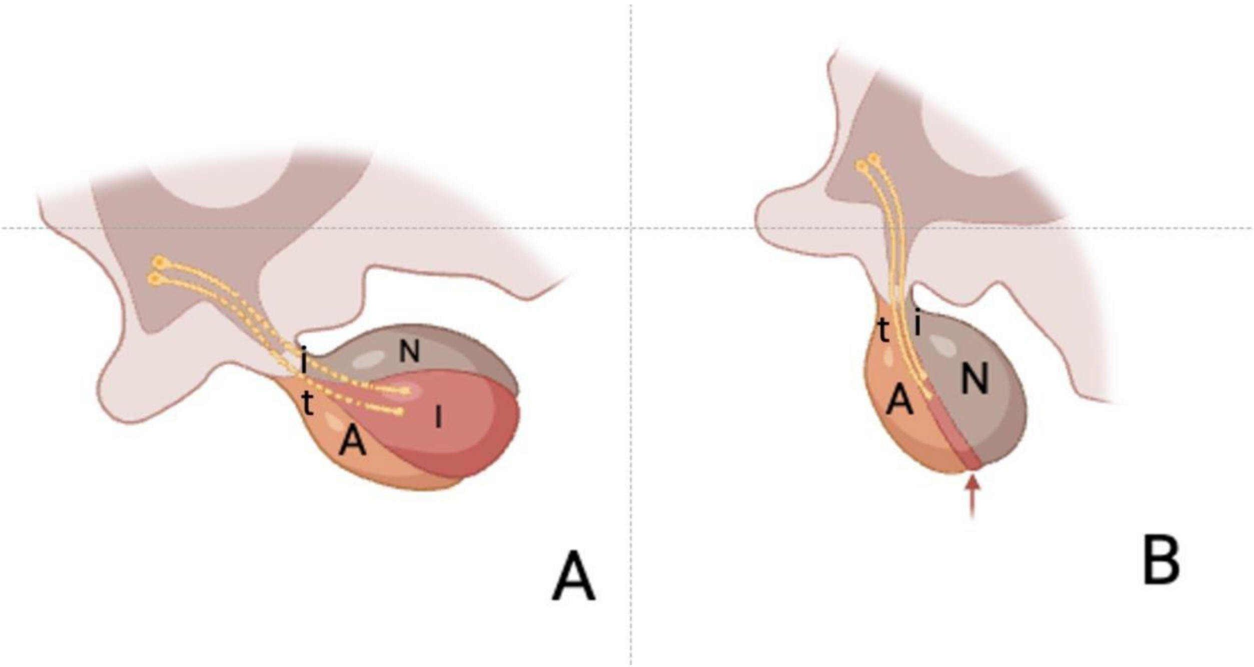

Figure S1. Comparative schematic drawings of the pituitary gland (hypophysis) in most mammals and that of cetaceans. (A) General structure of the hypophysis in most mammals, showing the adenohypophysis or pars distalis (A) (A, neurohypophysis or pars nervosa (N), the intermediate lobe or pars intermedia (I), the infundibulum (i), and the pars tuberalis (t). (B) General structure of the hypophysis in cetaceans, where the pars intermedia is absent. The arrow indicates the separation between the adenohypophysis (A) and neurohypophysis (N) by a dura mater recess. Abbreviations: i, infundibulum; t, pars tuberalis. Created in BioRender. Alonso, P. (2025) https://BioRender.com/b14i244

CET NUMBER	SAMPLE ID	Species	Sex	Age	Conservation code	Live/dead stranding	Skull Opening Protocol	Study
CET 1145	SA218/21	*Stenella frontalis*	male	calf	fresh	dead	Sacchini et al., 2022	Histology IHC
CET 1151	SA167/21	*Tursiops truncatus*	male	juvenile	fresh	dead	Sacchini et al., 2022	Histology
CET 1152	SA297/21	*Stenella frontalis*	male	calf	Very fresh	live	Sacchini et al., 2022	Histology IHC
CET 1153	SA305/21	*Delphinus delphis*	male	adult	fresh	dead	Sacchini et al., 2022	Histology
CET 1158	SA207/21	*Stenella frontalis*	male	adult	fresh	dead	Sacchini et al., 2022	Histology
CET 1161	SA217/21	*Stenella frontalis*	male	adult	fresh	dead	Sacchini et al., 2022	Histology
CET 1162	SA345/21	*Stenella frontalis*	female	juvenile	fresh	dead	Sacchini et al., 2022	Histology
CET 1163	SA411/21	*Stenella frontalis*	female	adult	fresh	dead	Sacchini et al., 2022	Histology
CET 1186	SA672/21	*Delphinus delphis*	female	adult	fresh	dead	Sacchini et al., 2022	Histology
CET 1200	SA1366/21	*Tursiops truncatus*	male	adult	fresh	dead	Sacchini et al., 2022	Histology
CET 1201	SA244/22	*Delphinus delphis*	female	adult	fresh	live	Sacchini et al., 2022	Histology
CET 1208	SA122/22	*Stenella frontalis*	female	adult	fresh	dead	Sacchini et al., 2022	Histology
CET 1212	SA177/22	*Stenella frontalis*	female	adult	fresh	dead	Sacchini et al., 2022	Histology
CET 1214	SA243/22	*Delphinus delphis*	female	adult	Very fresh	live	Proposed modification	Histology IHC TEM
CET 1217	SA335/22	*Stenella frontalis*	female	adult	fresh	dead	Sacchini et al., 2022	Histology
CET 1218	SA308/22	*Stenella frontalis*	male	calf	fresh	dead	Sacchini et al., 2022	Histology
CET 1220	SA405/22	*Stenella frontalis*	female	adult	fresh	dead	Proposed modification	Histology
CET 1221	SA334/22	*Delphinus delphis*	male	adult	fresh	live	Proposed modification	Histology
CET 1224	SA395/22	*Delphinus delphis*	male	adult	fresh	live	Sacchini et al., 2022	Histology IHC
CET 1232	SA930/22	*Delphinus delphis*	male	adult	fresh	live	Sacchini et al., 2022	Histology
CET 1239	SA696/22	*Tursiops truncatus*	male	juvenile	Very fresh	live	Proposed modification	Histology IHC
CET 1248	SA779/22	*Stenella frontalis*	male	neonate	fresh	dead	Sacchini et al., 2022	Histology
CET 1260	SA1539/22	*Delphinus delphis*	male	adult	Very fresh	live	Sacchini et al., 2022	Histology IHC TEM
CET 1261	SA1556/22	*Tursiops truncatus*	female	adult	fresh	dead	Proposed modification	Histology IHC
CET 1265	SA1661/22	*Stenella frontalis*	female	adult	fresh	live	Proposed modification	Histology IHC
CET 1270	SA225/23	*Stenella frontalis*	female	adult	fresh	dead	Proposed modification	Histology
CET 1279	SA331/23	*Stenella frontalis*	female	adult	fresh	dead	Sacchini et al., 2022	Histology
CET 1287	SA380/23	*Stenella frontalis*	female	juvenile	fresh	live	Proposed modification	MRI Histology IHC
CET 1294	SA463/23	*Delphinus delphis*	female	neonate	fresh	live	Proposed modification	Histology
CET 1315	SA664/23	*Stenella frontalis*	female	adult	fresh	dead	Sacchini et al., 2022	Histology
CET 1324	SA270/24	*Stenella*	female	adult	fresh	live	Proposed	Histology

		frontalis					modification	
CET 1333	SA368/24	*Stenella frontalis*	female	adult	fresh	live	Sacchini et al., 2022	Histology
CET 1334	SA328/24	*Stenella frontalis*	male	adult	fresh	live	Proposed modification	Histology IHC
CET 1337	SA451/24	*Delphinus delphis*	male	juvenile	fresh	dead	Proposed modification	Histology IHC TEM
CET 1339	SA476/24	*Stenella frontalis*	male	adult	fresh	live	Proposed modification	Histology
CET 1343	SA436/24	*Stenella frontalis*	male	adult	fresh	live	Proposed modification	Histology
CET 1345	SA481/24	*Delphinus delphis*	female	adult	fresh	dead	Proposed modification	Histology
CET 1350	SA523/24	*Delphinus delphis*	female	adult	fresh	dead	Proposed modification	Histology
CET 1355	SA569/24	*Stenella frontalis*	male	adult	fresh	dead	Proposed modification	Histology
CET 1358	SA614/24	*Stenella frontalis*	male	adult	fresh	dead	Proposed modification	Histology
CET 1371	SA862/24	*Tursiops truncatus*	male	adult	fresh	live	Proposed modification	Histology
CET 1380	SA1194/24	*Delphinus delphis*	female	adult	fresh	dead	Proposed modification	Histology
NA	SA244/20	*Tursiops truncatus*	male	calf	Very fresh	NA	Sacchini et al., 2022	Histology IHC
NA	SA1037/21	*Tursiops truncatus*	male	adult	Very fresh	NA	Sacchini et al., 2022	Histology IHC
NA	SA540/21	*Tursiops truncatus*	male	adult	Very fresh	NA	Sacchini et al., 2022	Histology IHC
NA	SA451/21	*Tursiops truncatus*	female	adult	Very fresh	NA	Proposed modification	Histology IHC
NA	SA135/23	*Tursiops truncatus*	male	neonate	Very fresh	NA	Proposed modification	Histology IHC

Table S1. Summary of stranded cetacean specimens included in this study. The table lists the unique cetacean identification number (CET NUMBER) and corresponding sample ID (SAMPLE ID) for each individual that indicates the year they stranted. The species are represented by Stenella frontalis (Atlantic spotted dolphin), Tursiops truncatus (bottlenose dolphin), and Delphinus delphis (common dolphin). Sex, age class (calf, neonate, juvenile, or adult), and conservation code (all fresh or very fresh) are provided. The "Live/dead stranding" column indicates whether the individual was found alive or dead when it stranded, in cases where the animal belonged to facilities under human care and did not strand this section was filled as NA (Not Applicable). The "Skull Opening Protocol" column indicates if either the protocol by Sacchini et al., (2022) was followed or the proposed modification presented in this work as one of the main methodological objectives. the protocol followed for skull dissection, either from Sacchini et al. (2022) or a proposed modification. Individuals without a CET NUMBER (NA; Not Applicable) correspond to the cases of common bottlenose dolphins under human care.

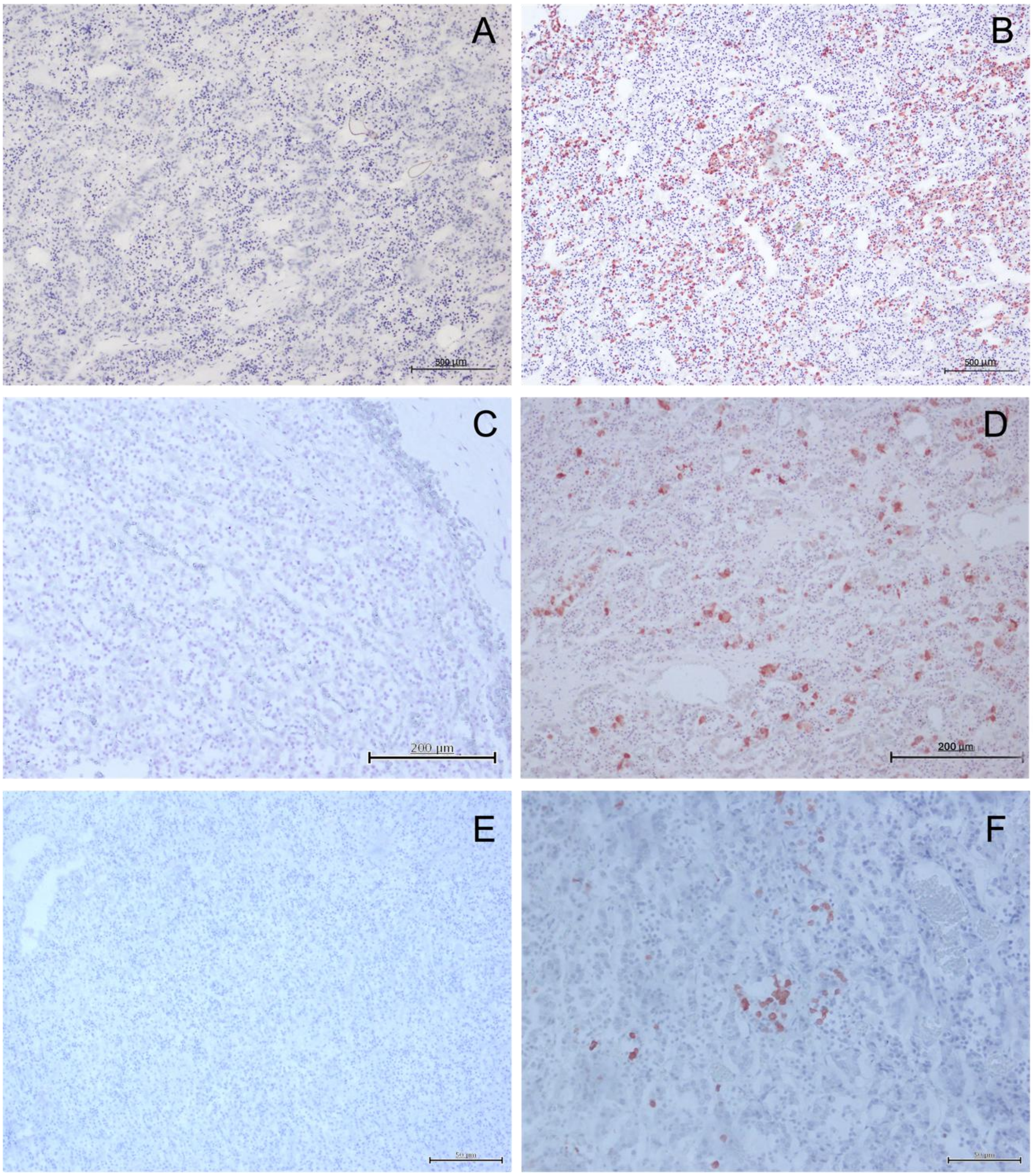

Figure S2. Positive and negative controls for immunohistochemistry (IHC) staining.
(A) Negative control for ACTH, where the primary antibody was omitted and replaced with nonimmune homologous serum in PBS, showing no specific staining. (B) Positive control for ACTH, using chimpanzee hypophysis tissue, displaying specific immunostaining. (C) Negative control for α-MSH, with the primary antibody omitted, showing no specific staining. (D) Positive control for α-MSH, showing specific immunostaining in chimpanzee hypophysis tissue. (E) Negative control for TSH, where the primary antibody was omitted, resulting in no specific staining. (F) Positive control for TSH, demonstrating specific immunostaining in chimpanzee hypophysis tissue. Counterstaining was performed with Mayer's Hematoxylin

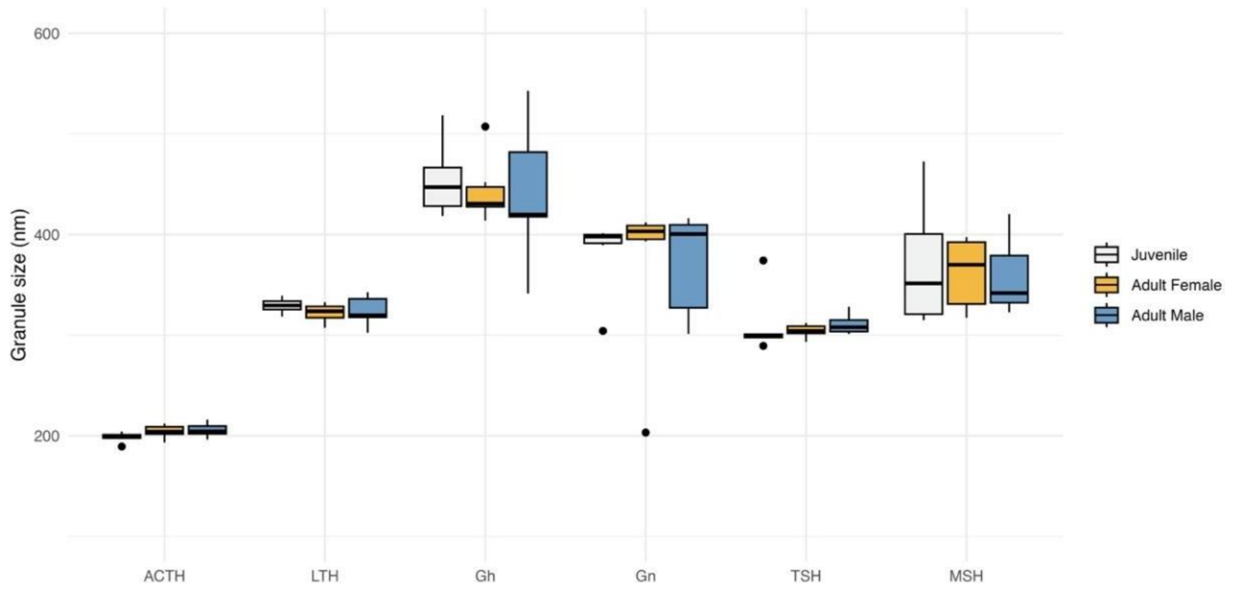

Figure S3. Average granule size for the different hormone-producing cells in the adenohypophyseal parenchyma of the common dolphins (*Delphinus delphis*) studied under Transmission Electron Microscopy. Granule sizes are presented by different age and sex groups. ACTH: corticotrophs; LTH: Lactotrophs; Gh (somatotrophs); Gn (gonadotrophs); TSH (thyrotrophs); MSH (melanotrophs).

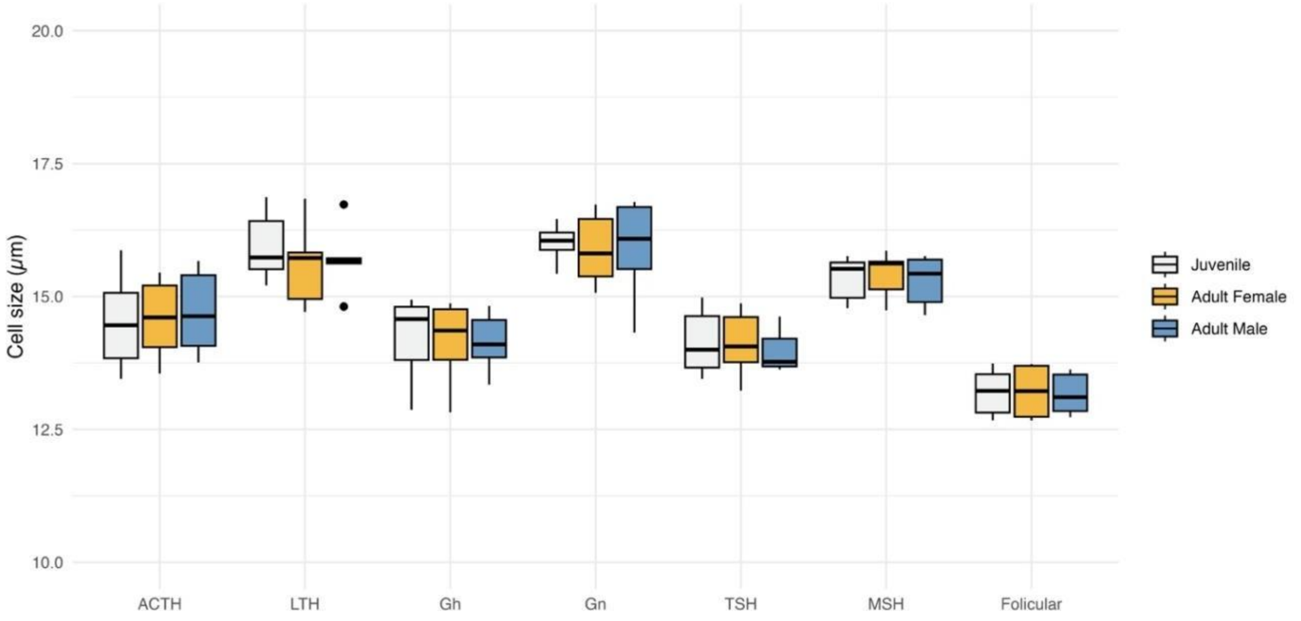

Figure S4. Average cell size for the different adenohypophyseal cells in common dolphins (*Delphinus delphis*) studied under Transmission Electron Microscopy. Cell sizes are presented by different age and sex groups. ACTH: corticotrophs; LTH: Lactotrophs; Gh (somatotrophs); Gn (gonadotrophs); TSH (thyrotrophs); MSH (melanotrophs).

Supplementary Document 1 – Administrative authorization For the handling of stranded specimens on the Canary Islands coast, the collection and custody of their biological samples, and the performance of necropsies for scientific and conservation purposes.

Summary of ethical approvals and permissions for this study. The document provides details of the administrative authorization issued by the Ministerio para la Transición Ecológica y el Reto Demográfico (MITECO), including the protocol number, date of approval, and issuing authority. A full copy of the authorization document is included below.

Ref: SGBTM/BDM/AUTSPP/72/2021

AUTORIZACIÓN ADMINISTRATIVA
PARA EL MANEJO DE EJEMPLARES VARADOS EN EL LITORAL DE CANARIAS, LA TOMA Y CUSTODIA DE SUS MUESTRAS BIOLÓGICAS Y LA REALIZACIÓN DE NECROPSIAS, CON FINES CIENTÍFICOS Y DE CONSERVACIÓN

El Instituto Universitario de Sanidad Animal y Seguridad Alimentaria (IUSA) de la Universidad de Las Palmas de Gran Canaria (ULPGC) viene realizando, desde hace más de 20 años, el estudio, monitorización y vigilancia sanitaria de la fauna marina a través de la realización de necropsias y de estudios de la causa de la muerte de los ejemplares varados en las costas de las islas Canarias. Este trabajo, encomendado por el Gobierno de Canarias, ha dado lugar a un valioso y abundante conocimiento científico sobre las poblaciones de cetáceos del archipiélago, que ha sido utilizado para la puesta en marcha de medidas de gestión para la conservación de estas especies.

El Centro Atlántico de Investigación de Cetáceos del IUSA-ULPGC -a través de la Fundación Canaria Parque Científico Tecnológico (FCPCT)- tiene previsto continuar con estos trabajos de apoyo a la red de varamientos de las islas Canarias. Para ello, cuanta con sus propias instalaciones y equipamiento, y tiene previsto reforzar la vigilancia sanitaria de las poblaciones de cetáceos y ampliarla a otras especies de fauna marina amenazada, asegurando la recogida de información científica, la toma de muestras biológicas y la realización de necropsias, que contribuyan eficazmente a un diagnóstico de salud de las especies marinas varadas y/o a la determinación de las posibles causas de muerte. Las muestras recogidas contribuirán a la mejora y ampliación del banco de tejidos y las bases de datos de fauna marina amenazada de las islas Canarias del IUSA- ULPGC.

Entre las tareas a desarrollar en el marco de estos trabajos se prevé la recogida, el transporte, la toma y custodia de muestras, de ejemplares varados muertos en las costas canarias, así como la realización de necropsias, de estudios histopatológicos y de otros análisis de laboratorio.

Estos trabajos con fines de conservación implican el manejo, el transporte, la toma y custodia de muestras y la realización de necropsia, de ejemplares de especies marinas protegidas varadas muertas, principalmente tortugas marinas, cetáceos y condrictios. En la mayoría de los casos, estas especies se encuentran protegidas por la legislación nacional e internacional, estando incluidas en el Anexo V de *Especies animales y vegetales de interés comunitario que requieren una protección estricta* de la *Ley 42/2007, de 13 de diciembre, del Patrimonio Natural y de la Biodiversidad*, e incluidas, además, en el *Listado de Especies Silvestres en Régimen de Protección Especial* o en el *Catálogo Español de Especies Amenazadas* (CEEA), ambos listados desarrollados por el *Real Decreto 139/2011, de 4 de febrero*. El resto de especies se encuentran protegidas por el artículo 54.5 sobre *Garantía de conservación de especies autóctonas silvestres* de la *Ley 42/2007, de 13 de diciembre*.

FIRMADO
JAVIER PANTOJA TRIGUEROS - 2021-11-22 20:04:23 CET, accion=Firma, cargo=Subdirector General de Biodiversidad Terrestre y Marina, unidad=DG Biodiversidad, Bosques y Desertificación, organizacion=Ministerio para la Transición Ecológica y el Reto Demográfico
La autenticidad del documento puede ser comprobada mediante el CSV: OIP_MSM7XXRQLQWX3AT4S9D2KRHDBWGI en https://www.pap.hacienda.gob.es

ESTA SUBDIRECCIÓN GENERAL, en virtud de la competencia estatal sobre biodiversidad marina establecida en el artículo 6 de la *Ley 42/2007, de 13 de diciembre, del Patrimonio Natural y de la Biodiversidad*, conforme a los artículos 61.1.d y 61.1.g de dicha ley, al artículo 3.4 de la *Ley 41/2010, de 29 de diciembre, de protección del medio marino,* y al 3.4 del *Real Decreto 1727/2007, de 21 de diciembre, por el que se establecen medidas de protección de los cetáceos*, y en virtud de las facultades que le han sido conferidas por el *Real Decreto 500/2020, de 28 de abril, por el que se desarrolla la estructura orgánica básica del Ministerio para la Transición Ecológica y el Reto Demográfico y Orden TED/533/2021, de 20 de mayo, sobre delegación de competencias,* ha resuelto:

AUTORIZAR

a D. Antonio Jesús Fernández Rodríguez (DNI 42159081-N), a D. Manuel Antonio Arbelo Hernández (DNI 42874415-T), a Dª. Eva María Sierra Pulpillo (DNI 30818220-Z), a D. Miguel Antonio Rivero Santana (DNI 43751421-Q), a Dª. María José Caballero Cansino (DNI 52483917-X), y a D. Jorge Francisco González Pérez (DNI 42849401-X), para llevar a cabo los trabajos descritos en las aguas bajo soberanía o jurisdicción española de la demarcación marina canaria, que implican el manejo, el transporte, la toma y custodia de muestras y la necropsia de animales marinos varados muertos y, en su caso, el acercamiento a ejemplares de diferentes especies de cetáceos y la estancia dentro del *Espacio Móvil de Protección de Cetáceos*, con los siguientes requerimientos:

- el ámbito geográfico de los trabajos se circunscribirá al litoral de Canarias;
- las principales especies objetivo son tortugas marinas, cetáceos, y condrictios;
- serán de obligado cumplimiento las medidas generales (art. 4) y complementarias de protección (art. 5) y las normas de conducta (Anexo II) establecidas en el *Real Decreto 1727/2007, de 21 de diciembre*, con las siguientes excepciones:
  - maniobras de aproximación a menos de 60 m de un cetáceo o grupo de cetáceos;
- cuando se estime necesario, se realizarán las correspondientes necropsias y/o recogida de muestras biológicas a los animales varados muertos y, si fuera necesario, la toma de muestras de tejido (piel) o sangre en animales vivos;
- se garantizarán unas condiciones de conservación óptimas de las muestras biológicas;
- se contará con un equipo cualificado y especializado en sanidad animal y/o patología de fauna marina, compuesto por licenciados en Veterinaria colegiados;
- las necropsias a ejemplares muertos serán realizadas por personal experto según el procedimiento estándar de ACCOBAMS y ASCOBANS *Best practice on cetacean post mortem investigation and tissue sampling*;
- los trabajos serán coordinados por el responsable del servicio, D. Antonio Jesús Fernández Rodríguez;
- para la recogida y traslado de animales y/o de muestras biológicas se utilizará un vehículo isotermo;
- para el desarrollo de los mencionados trabajos se antepondrá en todo momento el bienestar animal.

FIRMADO
JAVIER PANTOJA TRIGUEROS - 2021-11-22 20:04:23 CET, accion=Firma, cargo=Subdirector General de Biodiversidad Terrestre y Marina, unidad=DG Biodiversidad, Bosques y Desertificación, organizacion=Ministerio para la Transición Ecológica y el Reto Demográfico
La autenticidad del documento puede ser comprobada mediante el CSV: OIP_MSM7XXRQLQWX3AT4S9D2KRHDBWGI en https://www.pap.hacienda.gob.es

En todo caso, se deberá contar y/o cumplir con las correspondientes licencias, trámites, obligaciones y autorizaciones de orden administrativo, fiscal, sanitario o laboral, exigidos por la legislación vigente, así como el correspondiente permiso de acceso a terrenos de titularidad privada y pública de otras instituciones.

Cuando la finalidad de la recolección de las muestras biológicas fuera el acceso para utilización de recursos genéticos, podrá ser de aplicación el ***Real Decreto 124/2017, de 24 de febrero, relativo al acceso a los recursos genéticos procedentes de taxones silvestres y al control de la utilización***. Por utilización se entenderá lo definido en el artículo 2.5 de dicha norma.

Si las actividades para las que se recogen las muestras biológicas no constituyen utilización, pero en el transcurso de las mismas deviene el interés para la utilización de recursos genéticos, previamente a su utilización, se deberá solicitar una autorización de acceso para aquellos recursos que se prevea utilizar conforme a lo estipulado en el *Real Decreto 124/2017, de 24 de febrero*.

No se podrán facilitar estas muestras biológicas a ninguna persona no autorizada y, en todo caso, la transmisión de las muestras biológicas a terceros se realizará en las mismas condiciones que las impuestas en esta autorización. En particular, si la cesión a terceros tiene como finalidad la utilización de recursos genéticos, podrá requerirse una autorización de acceso a recursos genéticos en virtud del *Real Decreto 124/2017, de 24 de febrero*.

La presente autorización será válida desde su firma y **hasta el 31 de diciembre de 2025**.

Deberá remitirse a la Subdirección General de Biodiversidad Terrestre y Marina (bzn-biomarina@miteco.es)), un informe anual con los resultados obtenidos en las actuaciones desarrolladas amparándose en la presente autorización, que incluya un archivo Excel con la siguiente información:

- datos anuales de toma de muestras biológicas (número de ejemplares, especie, edad y sexo, tipo de muestra, banco de destino de las muestras, transporte y cadena de custodia, en su caso);
- datos anuales de necropsias realizadas (especie, número de ejemplares, edad y sexo, causa probable de la muerte, presencia de plásticos en el tracto digestivo, etc.);
- en su caso, actividades realizadas en el *Espacio Móvil de Protección de Cetáceos:* distancia a los cetáceos o grupo de cetáceos a la que se ha llegado en cada caso y tiempo de permanencia;
- destino dado a las muestras y restos recogidos;
- actualización de la relación de material de muestras que posea el IUSA-ULPGC;
- descripción de los diferentes trabajos e incidencias, en su caso:
- resultado de los análisis y estudios realizados;

FIRMADO
JAVIER PANTOJA TRIGUEROS - 2021-11-22 20:04:23 CET, accion=Firma, cargo=Subdirector General de Biodiversidad Terrestre y Marina, unidad=DG Biodiversidad, Bosques y Desertificación, organizacion=Ministerio para la Transición Ecológica y el Reto Demográfico
La autenticidad del documento puede ser comprobada mediante el CSV: OIP_MSM7XXRQLQWX3AT4S9D2KRHDBWGI en https://www.pap.hacienda.gob.es

Los datos que se incluyan en este informe deberán presentarse en el formato que se determine por la Subdirección General de Biodiversidad Terrestre y Marina, de manera que puedan integrarse en el Sistema Integrado de Información del Banco de Datos de la Naturaleza.

Asimismo, deberá remitirse a la Subdirección General de Biodiversidad Terrestre y Marina (bzn-biomarina@miteco.es) copia de cualquier material científico-técnico y educativo desarrollado amparándose en la presente autorización. En todo caso, para la publicación o divulgación, por cualquier medio de comunicación, de información relativa al proyecto y/o de imágenes tomadas durante el mismo, **se deberá señalar que esta ha sido obtenida tras solicitar autorización del Ministerio para la Transición Ecológica y el Reto Demográfico**.

El incumplimiento de los preceptos indicados en esta autorización que puedan infringir lo establecido en la materia en la *Ley 42/2007, de 13 de diciembre*, podrá generar responsabilidad de naturaleza administrativa, sin perjuicio de la exigible en vía penal, civil o de otro orden en que puedan incurrir. La inobservancia de cualquiera de los preceptos indicados anteriormente podrá suponer la revocación de la presente autorización y la no autorización de nuevas solicitudes.

En el ámbito de la Administración General del Estado, contra la resolución de la presente autorización podrá interponerse recurso de alzada ante el Secretario de Estado de Medio Ambiente en el plazo de un mes de acuerdo con lo previsto en los artículos 121 y 122 de la *Ley 39/2015, de 1 de octubre.*

EL SUBDIRECTOR GENERAL
DE BIODIVERSIDAD TERRESTRE Y MARINA

(Firma electrónica)

Javier Pantoja Trigueros

FIRMADO

JAVIER PANTOJA TRIGUEROS - 2021-11-22 20:04:23 CET, accion=Firma, cargo=Subdirector General de Biodiversidad Terrestre y Marina, unidad=DG Biodiversidad, Bosques y Desertificación, organizacion=Ministerio para la Transición Ecológica y el Reto Demográfico
La autenticidad del documento puede ser comprobada mediante el CSV: OIP_MSM7XXRQLQWX3AT4S9D2KRHDBWGI en https://www.pap.hacienda.gob.es
